# Oral neutrophils are an independent marker of the systemic inflammatory response after cardiac bypass

**DOI:** 10.1186/s12950-014-0032-5

**Published:** 2014-10-18

**Authors:** Mary Elizabeth Wilcox, Emmanuel Charbonney, Pablo Perez d’Empaire, Abhijit Duggal, Ruxandra Pinto, Ashkan Javid, Claudia Dos Santos, Gordon David Rubenfeld, Susan Sutherland, Wayne Conrad Liles, Michael Glogauer

**Affiliations:** Interdepartmental Division of Critical Care, University Health Network, Toronto, Canada; Division of Critical Care, Department of Medicine, University of Montreal, Montreal, Canada; Interdepartmental Division of Critical Care, Trauma, Emergency and Critical Care Program, Sunnybrook Health Sciences Centre, Toronto, Canada; Faculty of Dentistry, University of Toronto, Matrix Dynamics Group, Toronto, Canada; Interdepartmental Division of Critical Care, St. Michael’s Hospital, Keenan Research Centre of Li Ka Shing Knowledge Institute, Toronto, Canada; Department of Dentistry, Sunnybrook Health Sciences Centre, Toronto, Canada; Division of Allergy and Infectious Disease, University of Washington, Seattle, USA

**Keywords:** Biomarkers, Multiple organ failure, Coronary bypass, Critical illness, Neutrophil activation

## Abstract

**Background:**

Cardiopulmonary bypass (CPB) is an immuno-reactive state where neutrophils are activated and accumulate in different tissues. Edema and tissue necrosis are the most common sequelae observed, predominantly in the lungs, kidneys, and heart, heralding significant risk for postoperative complications. No method exists to noninvasively assess in vivo neutrophil activity. The objective of this study was to determine if neutrophil recruitment to the oral cavity would correlate with specific biomarkers after coronary bypass surgery (CPB).

**Methods:**

We conducted a single site prospective observational study including non-consecutive adult patients undergoing elective, on-pump CPB. Blood and either oral cavity rinses or swabs were collected pre- and post-CPB. Absolute neutrophil counts from oral samples and serum biomarkers were measured. The association between neutrophil recruitment to the oral cavity, biomarkers and outcomes after CPB were analyzed.

**Results:**

CPB was associated with statistically significant increases in oral and blood neutrophil counts, as well as an increase in certain biomarkers over preoperative baseline. Peripheral blood neutrophil count were increased at all time points however statistically significant differences in median oral neutrophil counts were observed only at the time point immediately postoperative, and in what seems to be two unique patient populations (p < 0.001; group 1, median: 1.6×10^5^, Interquartile range [IQR], 1.1×10^5^ - 4.8×10^5^, and group 2, median: 1.9×10^6^, IQR, 8.7×10^5^ - 4.0×10^6^).

**Conclusions:**

CPB is associated with a transient increase in oral neutrophils that may correlate with the systemic inflammatory response; oral neutrophils may have the ability to discriminate and identify unique patient populations based on their tissue migration.

## Introduction

The innate immune system provides the host with an immediate but non-specific response to infection. Although inflammation is an essential response, its onset and progression may become “dysregulated”, resulting in a massive and uncontrolled release of proinflammatory mediators that may lead to widespread tissue injury [1]. The systemic inflammatory response system (SIRS) is a complex clinical syndrome involving complement activation, cytokines, coagulation, fibrinolytic and kallikrein cascades. Neutrophils play a pivotal role in defense as they contain and destroy causative organisms after breaching host anatomical barriers, such as the skin and mucosa. Neutrophil-mediated organ dysfunction has been implicated as playing a causative role in the high rates of morbidity and mortality in SIRS patients [2].

Coronary artery bypass grafting (CABG) is a well-established treatment for ischemic heart disease. It is traditionally performed using cardiopulmonary bypass (CPB), termed “on-pump CABG”. CPB is an immuno-reactive state, where polymorphonuclear (PMN) cells are activated through contact with the synthetic surfaces of the bypass circuit, mechanical shear stress and hemodilution, [3-6] and as a result accumulate in different tissues. Nonrandomized studies have shown a marked difference in complement activation (C3a, C5a) between on-pump and off-pump CPB patients [7]. Similar such studies have shown a significant and persistent increase in lymphocytes and neutrophils postoperatively in on-pump CPB patients when compared to off-pump CPB patients [8]. This widespread PMN activation generates an abundance of oxygen and hydroxyl free radicals that can exacerbate the release of granular constituents such as elastase and myeloperoxidase into surrounding tissues. Edema and tissue necrosis are the most common sequelae observed predominantly in the lungs, kidneys and heart heralding significant risk for postoperative complications [9,10]. Examination of autopsy specimens from patients with multiple organ failure (MOF) revealed localization of neutrophils in both renal blood vessel aggregates and large-scale infiltration of the lung tissues [11]. In acute lung injury, the intensity of neutrophil infiltrates correlates with impaired lung function and with high concentrations of neutrophil-derived proteolytic enzymes in bronchoalveolar lavage [12].

Currently there is no rapid non-invasive method of assessing in vivo neutrophil function as a surrogate of an individual’s innate immune function. The standard routine measure for assessing a patient’s innate immune status involves monitoring circulating PMN levels. While this is an informative clinical parameter as it relates to cell numbers, it provides no information about cell functionality or the efficiency with which these cells reach the site of infection and eliminate microbial burden. The primary role of a neutrophil is to eliminate microbes at sites distant from the circulation. While monitoring neutrophil recruitment to tissues following CPB may be beneficial in determining those patients who are more susceptible to CPB-related morbidity, tissue biopsy is invasive and impractical.

Research suggests that the oral cavity may provide an early opportunity to non-invasively monitor the innate immune system [13-15]. The oral cavity is optimal for this as it contains an ever-present bacterial biofilm on teeth, tongue and other shedding surfaces. This bacterial presence is kept under control, in part, by the constant influx of neutrophils that migrate into the mouth from the periodontal tissues, which surround and support the teeth [16]. The recruitment of neutrophils by the oral biofilm may serve as a non-invasive measure of in vivo systemic neutrophil activation and chemotaxis [13-15].

The primary aim of this study was to show that CPB increases oral neutrophil counts, measured by oral rinses or swabs. The secondary aims were to demonstrate that CPB is associated with a change in oral neutrophil counts, independent of its effect on neutrophils in the peripheral circulation, and that changes are seen in levels of both pro- and anti-inflammatory biomarkers. Our hypothesis was that changes in oral neutrophil counts would correlate with specific biomarkers after CPB and possibly be associated with CPB-related morbidity. CPB induced changes in oral neutrophils could also demonstrate proof of concept for exploring oral neutrophil counts as marker for neutrophil activation in other inflammatory states seen in critical care.

## Methods

The Sunnybrook Health Sciences Centre Research Ethics Board approved this protocol. Written consent was provided by study participants.

### Study subjects

Adult patients (>18 years of age) undergoing elective, on-pump cardiopulmonary bypass surgery were enrolled in this study at Sunnybrook Health Sciences Centre following attainment of informed consent. Exclusion criteria included: existing oral disease (e.g. oral cancer, congenital or acquired chronic mucocutaneous disease), ongoing infection (e.g. endocarditis), recent myocardial infarct (i.e. within 2 weeks), pregnancy, pre-existence of any autoimmune or immune deficiency disease and/or patients who had been treated with steroids or other immunosuppressive agents within the previous 30 days.

### Sample collection and frequency

Either a brief oral rinse or swab was performed to collect neutrophils from the oral cavity. Oral rinses were not performed while a patient was intubated and mechanically ventilated due to the risk of aspiration. For the oral rinse sample, patients rinsed with 10 mLs of sterile normal saline (9%) for 30 seconds [14,17]. Samples for the oral swab, were obtained from four-quadrant gingival swabs [13]. Oral neutrophil samples were obtained from patients pre-operatively (T_0_) and postoperatively upon arrival to the CVICU (T_1_), at 12–18 hours (T_2_), and on day 3 (T_3_). All samples were stored at 4°C prior to transportation to the lab for analysis.

### Assessment of oral PMN levels

The protocol used to collect and count oral PMNs was a modification of previous work [18,19]. Each oral collection sample was collected in a sterile vial and stored at 4°C to preserve the cells prior to transportation to the laboratory for processing. All samples were processed within 24 hours of collection; the oral rinse sample underwent centrifugation at 2500 RPM for 5 minutes at 21°C (Hettich Rotina 35R, Rare Scientific, Edmonton, Canada). The cell pellet was re-suspended in 1 mL of double distilled water. Twenty milligrams of 2,2′-azino-bis(3-ethylbenzo-thiazoline-6-sulfonic acid) (ABTS; AO; Sigma Chemical, Burlington, Canada) was dissolved in 3.6 mL of 1 MM phosphocitrate buffer to produce a 1X concentrated solution. 45.6 μL of 30% hydrogen peroxide was added to 3.952 mL of double distilled water to produce a hydrogen peroxide homogenous solution. For each 1 mL concentrated oral rinse sample, 100 μL of the ABTS solution followed by 100 μL of 30% hydrogen peroxide solution was added in order to observe the characteristic blue-green colour change. After the colour reaction was complete, 250 μL of the sample was added to a 96 well plate in triplicate. The absorbance was measured at 420 nm for 10 cycles at 180 seconds per cycle using an automated microplate reader software (FLUOstar OPTIMA; BMG LABTECH GmbH, Offenburg, Germany). The final absorbance value for a sample was given as the average of the absorbance values of the cycles and of the triplicate measurements. The average standard deviation and coefficient of variation of the absorbance of the 10 cycles for all the collected samples was 0.09 and 6.34% respectively indicating a very low variation between the cycle absorbance readings. In order to determine the PMN counts within a given oral rinse sample based on the absorbance measure, a standard curve equation was used (y = 3305783.5X-1366). A series of standard solutions were prepared in the lab as described above, using neutrophils of known numbers/concentrations. The absorbance was then measured at 420 nm for 10 cycles at 180 seconds per cycle using the software (FLUOstar OPTIMA; BMG LABTECH GmbH, Offenburg, Germany). The standard curve equation was then obtained by plotting the absorbance of the standard solutions versus the known concentrations, thus allowing deduction of the PMN counts using the absorbance value obtained for any one sample.

### Blood neutrophil counts

Daily routine circulating white blood cell differential counts were used to compare with oral neutrophils. Values approximating most closely to the time of oral neutrophil collection were used. For patients that were still in hospital at day 7, the differential neutrophil count was used for comparative analysis.

### Biomarker analysis

All subjects received concurrent blood draws. Samples were drawn in ethylenediaminetetraacetic acid and citrate tubes, centrifuged at 1700 × 2 g at 4°C for 20 mins, plasma was collected and frozen in cryogenic tubes at −80°C within hours of collection. Plasma was assayed for Interleukin (IL)-1β, IL-2, IL-6, IL-10, IL-12, IL-17, IL-1RA, monocyte chemotactic protein (MCP)-1, soluble intercellular adhesion molecule (sICAM), granulocyte/macrophage colony-stimulating factor (GM-CSF) using a Luminex mediator panel with Multiplexing immunoassays instrument (Luminex technology, Austin, USA). All assays were performed in duplicate with the average level used in the analysis.

### Clinical parameters

Comprehensive clinical data was collected on the day of admission and through out the duration of the study, including: demographics, baseline characteristics (e.g. risk factors for coronary artery disease, medications, etc.), details of the surgical procedure and postoperative course (e.g. duration of mechanical ventilation, length of stay (LOS), documented infections (respiratory, urinary and catheter infections), and incidence of acute renal failure or need for renal replacement therapy (RRT).

### Statistical analysis

Continuous variables were summarized with means (standard deviation, SD) or medians and IQR and categorical variables were summarized using frequencies and percentages. The degree of association between oral neutrophil counts (swab and rinse) was determined using Spearman’s correlation [20]. Changes between preoperative and postoperative neutrophil counts were compared using Wilcoxon signed-rank test. To compare the changes in the oral swab between groups with low baseline oral neutrophils and high baseline neutrophils we used Wilcoxon rank-sum test. Association between change in oral neutrophil counts and measured cytokines in blood samples was compared using Spearman rank correlation coefficient (all values were transformed to a logarithmic scale for analysis). Linear regression was used to determine the association of measured covariates and change in pre- and postoperative oral neutrophil counts. Consideration for inclusion of a variable in the multivariable model required a p-value <0.2 in the univariable analysis. Differences were considered significant at p-value ≤0.05. All analyses were performed without adjusting for multiple-comparison using SAS 9.3 (SAS Institute Inc., Cary, NC, USA).

## Results

### Population characteristics

There were 41 patients enrolled with a median age of 67 years (IQR, 60–72), years; 78% were male; 76% were Caucasian; and there was a high co-morbid burden of disease: diabetes mellitus (24%), hypertension (73%), and renal disease (20%) (Table 1). CPB was indicated for 21 (51%) valve replacements and 20 (49%) vessel grafts: single vessel (10%), double bypass (20%), triple bypass (50%) and quadruple bypass (20%). The median length of time spent on bypass was 132 mins (IQR, 113–148 mins) and with the aorta cross-clamped 107 mins (IQR, 93–122 mins). Acute Physiology and Chronic Health Evaluation (APACHE) II and Multiple Organ Dysfunction Score (MODS) scores were 22 (SD ±4) and 6.2 (SD ±2.2), respectively on the day of admission (Table 1). Patients were mechanically ventilated for a median of one-day (IQR, 1–1 days) and median hospital length of stay was 8 days (IQR, 7–10 days). At the time of hospital discharge, 35 patients (85%) went home whereas the remaining patients went to a rehabilitation facility.

**Table 1:** **Baseline characteristics of patients in study (n = 41)**

**Characteristic**	**Mean (SD)**
Age – yrs	67 (60–72)
Male sex – no. (%)	32 (78)
Co-morbidities	
Diabetes Mellitus (requiring OHGs or insulin) – no. (%)	10 (24)
Smoking status reported as active – no. (%)	5 (12)
Hypertension	30 (73)
Renal insufficiency	8 (20)
Medication use – no. (%)	
Statin	22 (54)
NSAID	2 (5)
ACE Inhibitor	13 (32)
Details of Surgery	
CPB time – mins*	132 (113–148)
Cross-clamp time – mins*	107 (93–122)
Temperature (low) – degrees Celsius*	32.2 (31.6–33.1)
Blood transfusion within 0–6 hrs of CPB initiation	10 (24)
Postoperative course	
Delta creatinine (baseline – immediate postoperative; μmol/L)*	3 (–2,12)
Any renal replacement therapy – no. (%)	0 (0)
Documented infection in CVICU – no. (%)	1 (2)
Patients in receipt of antibiotic therapy – no. (%)	4 (10)
Severity of Illness	
APACHE II score	22 (4.4)
MODS postoperative day 0	6.2 (2.2)
MODS postoperative day 1	0.82 (1.5)
MODS postoperative day 2	0.63 (1.4)
MODS postoperative day 3	0.34 (1.2)
Ventilator use in days – no. (%)	
1 day	36 (88)
2 days	3 (7)
3 days	1 (2)
11 days	1 (2)
Length of stay in ICU – days*	2 (2–5)
Length of hospitalization*	8 (7–10)

*Median (IQR).

OHGs: oral hypoglycemic agents; NSAID: non-steroidal anti-inflammatory drug; ACE: angiotensin-converting-enzyme; CPB: cardiopulmonary bypass; CVICU: cardiovascular intensive care unit; APACHE: Acute Physiology and Chronic Health Evaluation; MODS: Multiple Organ Dysfunction Score.

### Oral neutrophil counts and CPB-associated inflammation

A significant correlation was seen between absolute oral neutrophil counts measured by oral swab as compared to oral rinse (rho = 0.83 [p < 0.001] and 0.70 [p < 0.001] for T_0_ and T_3_, respectively). No significant correlation was seen between absolute oral neutrophil counts as measured by oral swab and circulating neutrophils measured in the peripheral circulation at any time point (Table 2). A significant difference was seen in the median oral neutrophil counts (oral swab) between both T_0_ and T_1_ (1.0×10^6^; IQR, 3.9×10^5^ – 3.3×10^6^; p < 0.001) and between T_0_ and T_2_ (1.3×10^5^; IQR, −1.1×10^5^ – 5.9×10^5^; p = 0.03) but not between T_0_ and T_3_ (1.6×10^4^; IQR, −2.8×10^5^ – 2.3×10^5^; p = 0.95; Table 3). Similar results were found with the oral rinse. Median differences in neutrophil counts in the peripheral circulation were 8.3×10^9^ (IQR, 4.7×10^9^ – 1.1×10^10^), 6.7×10^9^ (IQR, 4.9×10^9^ – 1.1×10^10^), 4.7×10^9^ (IQR, 3.1×10^9^ – 7.1×10^9^), and 1.5×10^9^ (IQR, 8.0×10^8^ – 3.1×10^9^) between T_1_, T_2_, T_3_, T_7_ and T_0_, respectively. Significant increases were seen in the absolute neutrophil counts in the blood between T_0_ and all other time points, including day 7 (p < 0.001).

**Table 2:** **Correlation between neutrophils collected from the oral cavity and peripheral blood**

	**Oral neutrophil counts**		**Peripheral blood neutrophil counts**	
	**Correlation (95% CI)**	**p-value for null hypothesis rho = 0**	**Correlation (95% CI)**	**p-value for null hypothesis rho = 0**
Oral swab T_0_	0.83 (0.70, 0.91)	<0.0001	−0.01 (−0.32, 0.30)	0.95
Oral swab T_1_	NA	..	−0.04 (−0.35, 0.28)	0.81
Oral swab T_2_	NA	..	−0.01 (−0.32, 0.30)	0.94
Oral swab T_3_	0.70 (0.48, 0.83)	<0.0001	−0.11 (−0.41,0.21)	0.49

**Table 3:** **Wilcoxon signed rank test of delta neutrophils for before and after cardiopulmonary bypass**

**Comparison groups**	**Median**	**Interquartile range (IQR)**	**p-value**
Oral swab T_0_:T_1_	1.0 x 10^6^	3.9 x 10^5^ – 3.3 x 10^6^	< 0.001
Oral swab T_0_:T_2_	1.3 x 10^5^	−1.1 x 10^5^ – 5.9 x 10^5^	0.03
Oral swab T_0_:T_3_	1.6 x 10^4^	−2.8 x 10^5^ – 2.3 x 10^5^	0.95
Blood T_0_:T_1_	8.3 x 10^9^	4.7 x 10^9^ – 1.1 x 10^10^	< 0.001
Blood T_0_:T_2_	6.7 x 10^9^	4.9 x 10^9^ – 9.5 x 10^9^	< 0.001
Blood T_0_:T_3_	4.7 x 10^9^	3.1 x 10^9^ – 7.1 x 10^9^	< 0.001
Blood T_0_:T_7_	1.5 x 10^9^	8.0 x 10^8^ – 3.1 x 10^9^	< 0.001

We first looked at univariate associations between change in oral neutrophils immediately following CPB (T_0_ – T_1_) and age, body mass index, gender, smoking status (only 5 patients were active smokers), use of immune mediating medication (statins/NSAIDs/ACE inhibitors or plavix), preoperative chlorhexidine rinse, blood transfusion, intraoperative temperature low, CPB time, cross-clamp time, number of vessels bypassed, valve replacement, aortic graft (only 5 patients), delta creatinine and need for vasopressors. Need for intraaortic balloon pump (IABP) or renal replacement therapy and intra-operative solumedrol administration could not be considered as no patient received RRT, only one patient required IABP, and one patient received intraoperative solumedrol administration. The only variables with p-value <0.2 in the univariable analysis is preoperative chlorhexidine wash (p-value = 0.01) and therefore no multiple regression analysis was performed. The same variables were used in univariable analyses (t-test or Pearson’s correlation for continuous variables) for change in oral neutrophils between T_0_ and T_3_. The following variables had a p-value <0.2: gender (p = 0.032), use of an immune mediating medication (p = 0.14), blood transfusion (p = 0.06), valve replacement (p = 0.017), aorta graft (p = 0.1; only 5 patients in aorta graft), intraoperative temperature low (p = 0.08), and greater number of vessels bypassed (p = 0.001). We started with all variables in the multiple regression models and then used backward selection method; in the final model gender was removed as not significant and only number of vessels bypassed was left (p = 0.001).

**Figure 1:**
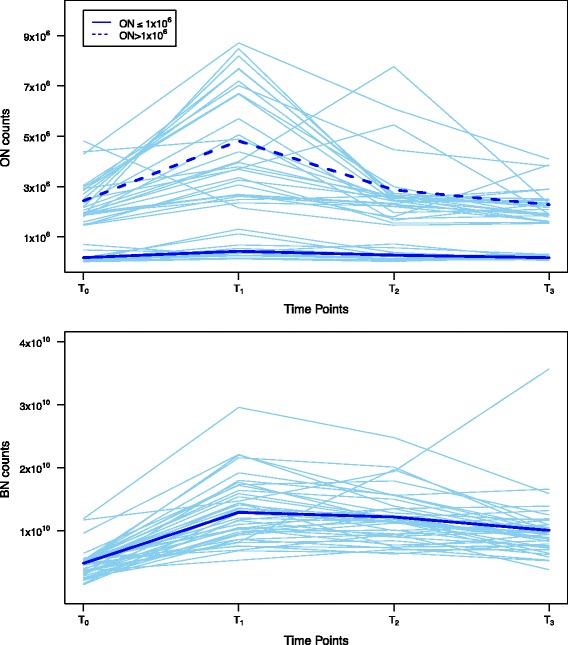
**Cardiopulmonary bypass increases both oral neutrophils (ON) [upper graph] and blood neutrophils (BN) [lower graph] counts at T**_**1**_ **.** At T_3_ compared to T_0_, the median oral neutrophil count was similar (p = 0.95), whereas median blood neutrophil count was significantly higher (p < 0.001).

Analysis of oral neutrophil counts identified two populations differentiated by their immediate response to CBP (difference between T_0_ and T_1_ oral neutrophil counts, Figure 1). Group 1 had baseline (T_0_) oral neutrophil counts ≤1×10^6^; and oral neutrophil counts did not significantly change after CPB (T_1_ ≤ 1.6×10^5^; IQR, 1.1×10^5^ – 4.8x10^5^). Group 2 had higher baseline oral neutrophil counts (T_0_ ≥ 1×10^6^) that increased significantly after CPB (T_1_ = 1.9×10^6^; IQR, 8.7×10^5^ – 4.0×10^6^; p-value = 0.0002). No statistically significant difference was seen between median oral neutrophil counts at any other time point between these patient groups (Table 4).

**Table 4:** **Wilcoxon rank-sum test of delta neutrophils for before and after cardiopulmonary bypass in two groups of patients (based on baseline oral neutrophil count)**

**Comparison groups**	**Median (IQR)**		**p-value**
	**Oral neutrophil counts T**_**0**_ **≤ 1x10** ^**6**^ **/μL**	**Oral neutrophil counts T**_**0**_ **≥ 1x10** ^**6**^ **/μL**	
Oral swab T_0_:T_1_	1.6x10^5^ (1.1x10^5^ – 4.8x10^5^)	1.9x10^6^ (8.7x10^5^ – 4.0x10^6^)	0.0002
Oral swab T_0_:T_2_	5.4x10^4^ (1.3x10^4^ – 2.7x10^5^)	2.7x10^5^ (−1.3x10^5^ – 7.0x10^5^)	0.40
Oral swab T_0_:T_3_	3.6x10^4^ (−3.9x10^4^ – 1.7x10^5^)	−4.4x10^4^ (−3.8x10^5^ – 3.7x10^5^)	0.90

### Biomarkers and CPB

**Figure 2:**
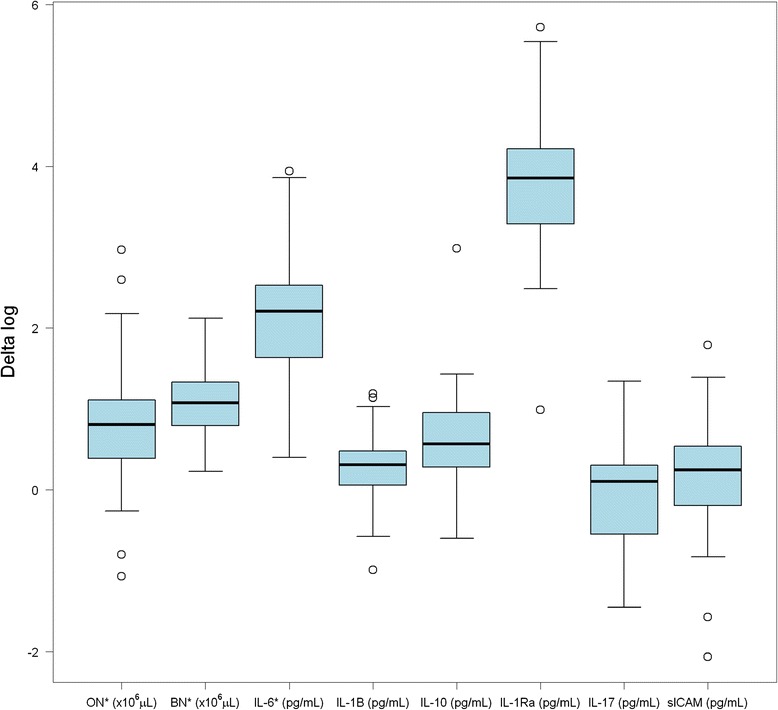
Cardiopulmonary bypass was associated with statistically significant increases in oral neutrophils and blood neutrophil counts, as well as certain biomarkers (*p < 0.05; T_**0**_ **vs. T**_**1**_ **).**

**Figure 3:**
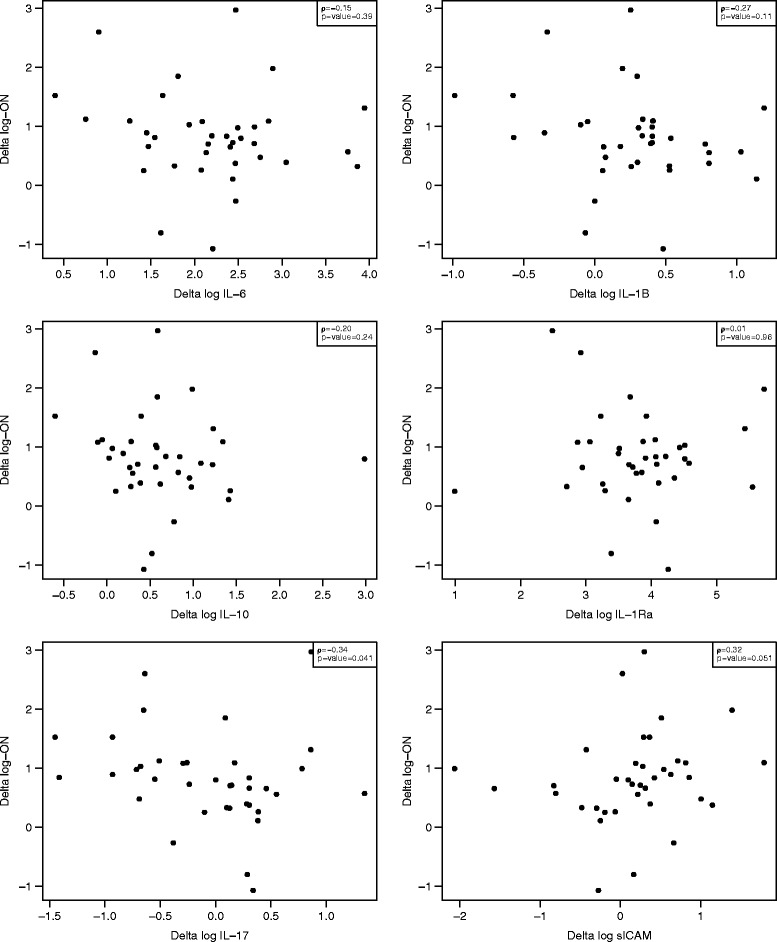
**Agreement between oral neutrophils and measured biomarkers: IL-17 and sICAM were statistically different from zero when compared before and immediately after cardiopulmonary bypass.**

Serum levels of IL-1β, IL-6, IL-10, MCP-1, IL-12 and IL-1RA significantly increased after CPB (T_0_ as compared to T_1_): mean difference, 1.45 ng/mL, 197.6 ng/mL, 28.2 ng/mL, 181.1 ng/mL, 9.1 ng/mL, and 4533.9 ng/mL, respectively. No significance difference in IL-2, IL-17, sICAM and GM-CSF levels were seen between T_0_ as compared to T_1_: mean difference, −1.0 ng/mL (p = 0.56), −1.3 ng/mL (p = 0.23), −3493.7 ng/mL (p = 0.42) and −6.56 ng/mL (p = 0.34) (p = 0.18; Figures 2 and 3).

### Neutrophil counts, biomarkers and severity of illness markers

All patients included survived to hospital discharge. Of special note, 3 (7%) of patients had a documented infection (clinical suspicion and positive cultures) and the mean change creatinine was 6 μmol/L (SD, ±17 μmol/L).

No significant correlations were seen between the change in oral or blood neutrophil counts (T_0_ as compared to T_1_) and the change in differences in the levels of any biomarker: IL-1β (rho = 0.25; p = 0.14), IL-2 (rho = 0.10; p = 0.55), IL-6 (rho = 0.09; p = 0.58), IL-10 (rho = −0.07; p = 0.69), IL-12 (rho = 0.14; p = 0.39), IL-17A (rho = −0.06; p = 0.73), MCP-1 (rho = 0.11; p = 0.53), IL-RA (rho = 0.31; p = 0.06), sICAM1 (rho = −0.01; p = 0.98) and GM-CSF (rho = 0.02; p = 0.93). Significant correlations however were seen between the change in log oral neutrophil counts (T_0_ as compared to T_1_) and the change in log levels of the biomarkers: IL-17 (rho = −0.34; p = 0.04), and sICAM (rho = 0.32; p = 0.05). The change seen in log blood neutrophils, comparing the same time points was found to negatively correlate with sICAM (rho = −0.41; p = 0.01).

Statistically significant positive correlations were seen in the differences in log oral neutrophil counts, T_0_ as compared to T_1_, and the MODS on days 1 (rho = 0.31; p = 0.05) and 3 (rho = 0.38; p = 0.01); no significant correlation was seen with MODS on day 0 or 2 (Table 5). Differences in log blood neutrophil counts, T_0_ as compared to T_1_, were negatively correlated with MODS on days 1 and 2 (rho = −0.44 and −0.32, respectively; p < 0.01 and 0.04). No significant correlation was seen with MODS on days 0 and 3; trends towards negative correlations (rho = −0.24 and −0.21, respectively). No significant correlation was seen between either differences in log oral or blood neutrophil counts, T_0_ as compared to T_1_, and change in creatinine levels (rho = 0.07; p = 0.69 and rho = −0.11; p = 0.48, respectively).

**Table 5:** **Spearman correlation coefficients and delta neutrophils (oral and circulating blood) for before and after cardiopulmonary bypass as compared with MODS on days 0 to 3**

**Comparison groups**	**Spearman correlation coefficient, (p-value for null hypothesis rho = 0)**			
	**MODS**	**MODS**	**MODS**	**MODS**
	**day 0**	**day 1**	**day 2**	**day 3**
Oral neutrophil counts T_0_:T_1_	0.17 (0.41)	−0.18 (0.26)	−0.08 (0.64)	0.10 (0.55)
Oral neutrophil counts T_0_:T_2_	−0.01 (0.97)	0.18 (0.27)	0.02 (0.20)	0.27 (0.09)
Oral neutrophil counts T_0_:T_3_	−0.23 (0.17)	0.09 (0.58)	0.14 (0.38)	0.06 (0.73)
Log oral neutrophil counts T_0_:T_1_	0.27 (0.09)	0.31 (0.05)	0.27 (0.09)	0.38 (0.01)
Log oral neutrophil counts T_0_:T_2_	0.13 (0.43)	0.35 (0.03)	0.28 (0.08)	0.39 (0.01)
Log oral neutrophil counts T_0_:T_3_	−0.14 (0.41)	0.11 (0.48)	0.12 (0.46)	0.10 (0.53)
Circulating blood neutrophil counts T_0_:T_1_	−0.17 (0.29)	−0.24 (0.13)	−0.21 (0.19)	−0.07 (0.65)
Circulating blood neutrophil counts T_0_:T_2_	−0.28 (0.08)	−0.14 (0.38)	0.01 (0.95)	−0.03 (0.83)
Circulating blood neutrophil counts T_0_:T_3_	−0.43 (0.01)	−0.20 (0.18)	−0.13 (0.40)	−0.14 (0.38)
Log circulating blood neutrophil counts T_0_:T_1_	−0.24 (0.13)	−0.44 (0.01)	−0.32 (0.04)	−0.22 (0.17)
Log circulating blood neutrophil counts T_0_:T_2_	−0.22 (0.17)	−0.25 (0.12)	−0.06 (0.69)	−0.14 (0.38)
Log circulating blood neutrophil counts T_0_:T_3_	−0.39 (0.01)	−0.25 (0.11)	−0.15 (0.35)	−0.12 (0.46)

## Discussion

Temporal difference between the mean blood and oral neutrophil counts in patients after CPB (a model of immune system activation) may predict vulnerability to CPB-related morbidity due to immune system dysregulation. The kinetics of delivery of neutrophils to tissues may be a more relevant determinant and marker of susceptibility to infection, and/or ongoing inflammation, than circulating white blood cell counts [11]. In this study, oral and blood neutrophil counts increased immediately after CPB, however oral neutrophil counts return to baseline by day 3 whereas peripheral circulating neutrophil counts remained (persistently) elevated at day 7. Associations between oral neutrophils and measured biomarkers IL-17 and sICAM before and immediately after CPB were found to be statistically significant. Interestingly, these were not the mediators that were most changed immediately after CPB but may provide the most relevant information regarding immune status [2,3,21].

Neutrophil activation is an integral component of the systemic host response. Neutrophils are the most abundant inflammatory cells, and their activation is essential for host defence against bacterial or fungal infection, as well as being principally involved in host injury in states of persistent inflammation. Neutrophil tissue repopulation following hematopoietic stem cell transplantation (HSCT) confers protection from infection [13]; whereas an excessive PMN response has previously been implicated in the pathogenesis of capillary leak syndromes resulting in decreased perfusion and end-organ damage, including acute respiratory distress syndrome and MOF [22-24]. The association of increased sICAM and IL-17 activity with an increase in the number of oral neutrophils suggests that oral neutrophil transmigration may be a viable surrogate marker of the acute pro-inflammatory immune response. IL-17, produced primarily by CD4+ Th17 cells, acts to enhance chemokine production by target cells in various tissues to promote/attract monocytes and neutrophils to those tissue (especially mucosal) sites, and in combination with sICAM suggests the predominance of a Th1 phenotypic response (acute pro-inflammatory state) [25-30]. IL-17 promotes endothelial activation by inducing the expression of endothelial adhesion markers (E-selectin, VCAM-1, and ICAM-1) in a p38 MAPK-dependent manner. This increased expression of adhesion molecules stimulates the trans-endothelial migration of neutrophils) [25-29]. Preventing neutrophil recruitment through blocking the action of IL-17 on endothelial cells (or on PMNs themselves) may prove to be highly beneficial in diseases in which neutrophilic inflammation plays a key role [25-30]. In animal models, reducing neutrophil invasion through IL-17A-blocking antibody has been shown to decreased infarct size and improved neurologic outcome in ischemic stroke [31] and has been shown to improve survival in endotoxic shock [32,33].

The timing of neutrophil recovery to the oral cavity following HSCT was recently found to have predictive value for future infections post-engraftment in a paediatric population [15] and be predictive of prognosis in an adult HSCT population [34]. Further, in a murine model the kinetics of neutrophil delivery to tissues during the engraftment phase was a better predictor of reconstitution of the immune system than reappearance of circulating neutrophils in the blood [35]. Neutrophil delivery to oral tissues has also been shown to predict success of HSCT at 6 months [34]. Neutrophil recovery from the oral cavity occurred more than one week earlier than that from the circulating blood and the time span between blood and oral neutrophil engraftment was inversely related to improved outcomes (i.e. longer delays between oral and blood engraftment predicted better outcome at 6 months) [34]. Should patients with persistent elevations in ON counts trend towards a worse outcome then early intervention (e.g. implementing a low tidal ventilation strategy) could decrease the pro-inflammatory cytokine response leading to end-organ failure [36,37]. It is important to note that there appears to be two patient populations based on the oral neutrophil levels. The first population has no change in their oral neutrophil levels from T_0_ to T_1_ where as a second population (the majority of the patients) did show an increase in that interval. This suggests that this test may have the ability to discriminate and identify unique patient populations based on neutrophil activation whereas the blood levels increased in all patients. One could hypothesize that whereas all patients had an increase of neutrophil levels in the circulation either through demargination or recruitment from the bone marrow, one subset of patients had neutrophils activated to the point where they were recruited into the oral cavity whereas a second smaller subset showed no such activation. A larger trial may be able to determine if the oral neutrophil clinical parameter has the sensitivity to identify patients at risk of poor outcomes.

Limitations of this study would include the small number of patients and the fact that none of the patients developed organ failure. The incidence of MOF from patients undergoing CPB is small (<5%) [38] and would necessitate a large patient sample to determine whether ON would predict an increase priming of PMNs and the development of MOF. However the number of patients in this study was chosen so as to have sufficient power to delineate differences in oral neutrophil counts, peripheral blood neutrophil counts and cytokine production based on previous CPB studies. Although in the present study no patients developed organ failure, the data suggests that these patients’ PMNs levels increased early following CPB, and therefore according to the two-hit model of MOF [10,39], were rendered vulnerable to end-organ dysfunction from such insults as post-operative bleeding or infection. Further, CPB was associated with an early, transient increase in oral neutrophil counts that correlated with the systemic inflammatory response as measured by certain cytokines and may be an additional marker of neutrophil or endothelial activation; identifying measures of oral neutrophil counts as a possible earlier indicator of a patient’s vulnerability to MOF. In addition, valuable information would be gathered by assessing neutrophil function, not just absolute number, through the measurement of myeloperoxidase activity. The local tissue inflammatory response could be assessed by measurement of local mediators. Given that this was a proof of concept study these investigations were not done. Strengths of this study would include demonstration of feasibility, that the oral rinse and swab provided similar results and therefore both techniques can be performed making these methods usable in critically ill patient population. Further, while the number of circulating PMNs seems to respond with relatively transient changes in immune status and seem to recover more slowly, it does not provide a functional assessment of PMN activity. Oral PMN counts are an established measure of functional activity; oral neutrophil counts measure PMN functional status as they reflect the ability of PMNs to transmigrate from the circulation to the mucosal surface [40-43]. Future studies will better investigate the relationship between morbidity and mortality in a critically ill patient population, with the goal of identifying patients at increased risk of poor outcomes.

## Conclusions

- sICAM and IL-17 activity is associated with increases in oral neutrophil number suggesting that oral neutrophil transmigration may be a surrogate marker of the acute pro-inflammatory immune response.
- Future studies should investigate the relationship between morbidity and mortality in a critically ill patient population and oral neutrophil transmigration, to identify patients at greatest risk of poor outcomes.
